# Anterior abdominal wall ‘peritoneal recess’: cause for pseudoherniation of small bowel resulting in chronic abdominal pain

**DOI:** 10.1308/003588412X13373405388211

**Published:** 2013-03

**Authors:** K Siddique, K Slaven, A Samad

**Affiliations:** St Helens and Knowsley Teaching Hospitals NHS Trust,UK

**Keywords:** Peritoneal recess, Pseudohernia, Chronic abdominal pain, Abdominal wall hernia

## Abstract

A middle-aged patient presented with intermittent chronic abdominal pain without any obvious cause. Computed tomography detected a hernia (presumed to be the cause of the patient’s symptoms) without any obvious lump on examination. A laparoscopy was performed to repair the hernia. This revealed a left-sided unilateral ‘peritoneal recess’ at the level of the arcuate line extending medial to the linea semilunaris. No extraperitoneal sac or defect was noted in the rectus sheath or in the muscle, nor were any contents present in the recess at the time of the laparoscopy. We believe the bowel was being trapped intermittently in this space, causing the abdominal symptoms.

A new potential space formed by a fold of the peritoneal layer of the anterior abdominal wall was found during a diagnostic laparoscopy performed to repair a possible anterior abdominal wall hernia. The suspected hernia was detected on computed tomography (CT) in a middle-aged patient presenting with intermittent abdominal pain without any obvious lump on examination (Fig 1). To our knowledge, this has not been reported before.

**Figure 1:**
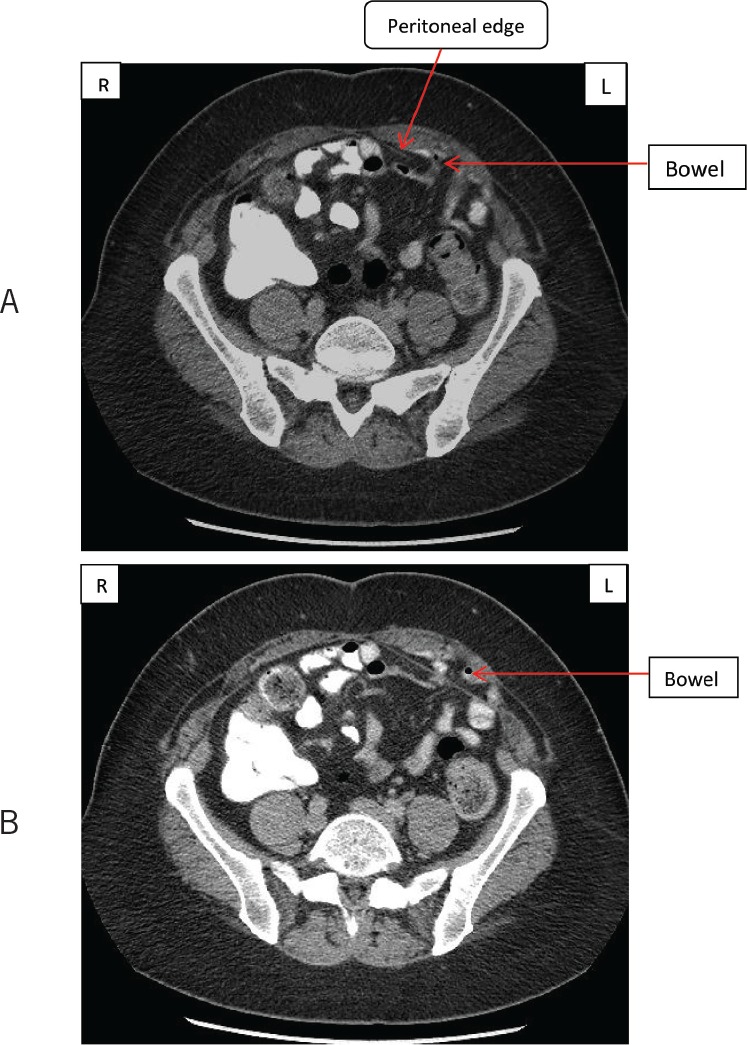
Computed tomography: the left-sided unilateral ‘peritoneal recess’ (A) and the bowel inside the peritoneal recess (arrow) (B)

At laparoscopy, a left-sided unilateral ‘peritoneal recess’ was noted at the level of the arcuate line extending medial to the linea semilunaris (Fig 2). The peritoneum was seen folded inwards subjacent to the posterior rectus sheath, conforming to the curve of the arcuate line for approximately 2.5cm, ending up in a blind recess (Fig 3). No extraperitoneal sac or defect was noted in the rectus sheath or in the muscle, nor were any contents present in the recess at the time of the laparoscopy. The peritoneal recess represented a potential space that corresponded well with the area on the CT showing small bowel herniation. We believe the bowel was being trapped intermittently in this space, causing the abdominal symptoms. By definition, this is not a true hernia and we would like to name this ‘Samad–Siddique’s pseudohernia’.

**Figure 2:**
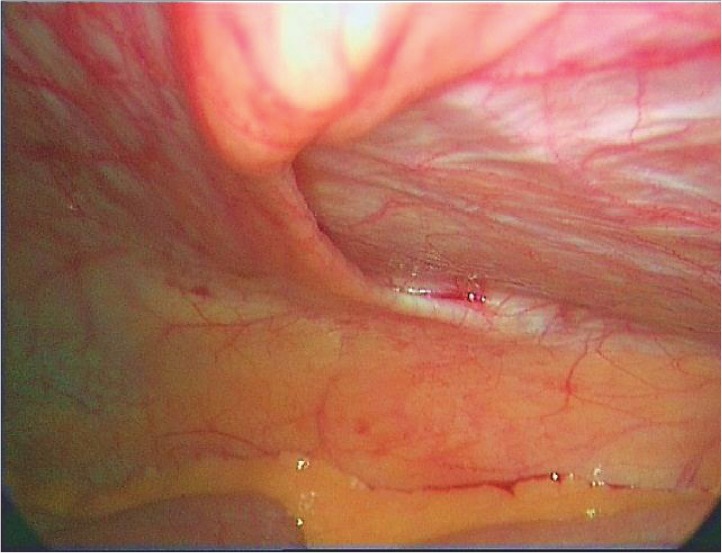
‘Peritoneal recess’ along the curve of left arcuate line

**Figure 3:**
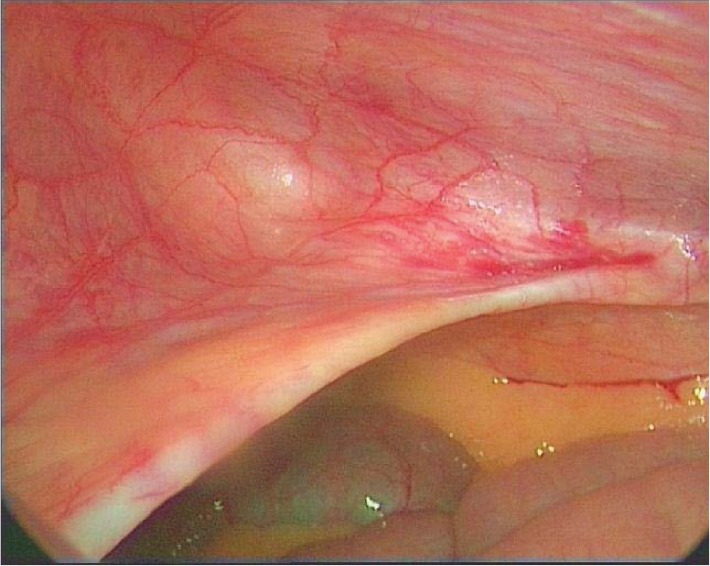
Extent of ‘peritoneal recess’

The boundaries (Fig 4) of the peritoneal recess are:

- *superiorly*: peritoneum subjacent to the posterior rectus sheath
- *inferiorly*: layers of peritoneal fold
- *apex*: junction of superior and inferior boundaries
- *lower limit*: peritoneal fold edge

**Figure 4:**
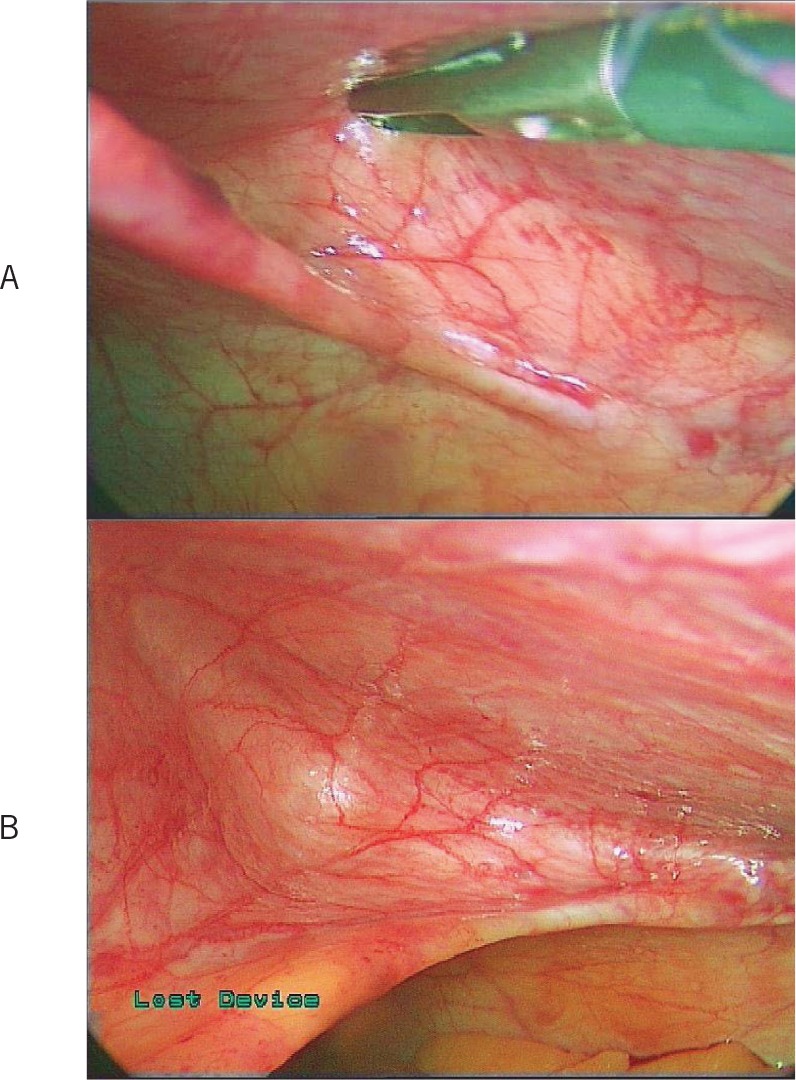
Boundaries of the ‘peritoneal recess’: recess opened up showing its apex (A) and superior, inferior and lower limits of recess (B)

The management of the peritoneal recess involves its closure with laparoscopic tackers along its entire length to prevent the bowel from further entering the recess, thereby resolving patient symptoms (Fig 5).

**Figure 5:**
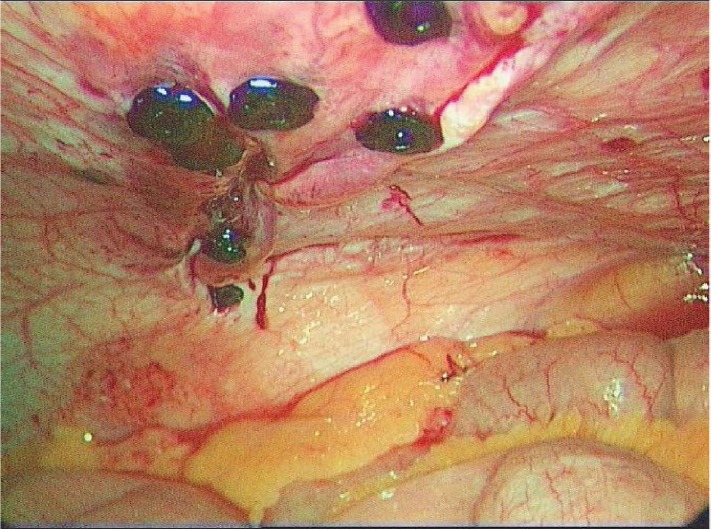
‘Peritoneal recess’ closed with endoscopic tackers

## Discussion

The new space reported here does not fall under the category of Spigelian hernia, interparietal hernia or superior hernia of the linea semilunaris. A Spigelian hernia has been described as a protrusion of the peritoneal sac, viscera or preperitoneal fat through a defect in the Spigelian aponeurosis.^1,2^ Interparietal hernias are very rare. The three subtypes are preperitoneal, interstitial and superficial. In the preperitoneal type, the hernia is between the peritoneum and transversalis fascia, interstitial type hernias lie between the muscle layers of the anterior abdominal wall, and in the superficial type it is between the external oblique and skin or within aponeuroses of the inguinal region.^3,4^ In the superior hernia of the linea semilunaris, the sac passes through the linea semilunaris at the level of the arcuate line to lie between the anterior layer of the internal oblique and the external oblique aponeurosis.^5^

## Conclusions

The ‘peritoneal recess’ is a potential space that neither penetrates through the posterior rectus sheath nor the rectus muscle. Anatomically, the hernia described here is a new variety of hernia that lies in a completely new location and has not been reported before. It does not therefore match any of the previously described types of hernia. We recommend a diagnostic laparoscopy for confirmation followed by endoscopic fixation of the recess.
